# (3,3′-{(1*E*,1′*E*)-1,1′-[Ethane-1,2-diylbis(azan-1-yl-1-yl­idene-κ*N*)]bis­(ethan-1-yl-1-yl­idene)}di­pyrazine 1-oxide-κ*N* ^4^)bis­(nitrato-κ*O*)nickel(II) monohydrate

**DOI:** 10.1107/S1600536813009355

**Published:** 2013-04-13

**Authors:** Mohammed A. S. Omer, Jia-Cheng Liu

**Affiliations:** a College of Chemistry and Chemical Engineering, Northwest Normal University, Lanzhou 730070, People’s Republic of China; b Department of Chemistry, Faculty of Education, University of Khartoum, Sudan

## Abstract

In the title complex, [Ni(NO_3_)_2_(C_14_H_16_N_6_O_2_)]·H_2_O, the Ni^II^ atom, lying on a twofold rotation axis, is coordinated by a tetra­dentate 3,3′-{(1*E*,1′*E*)-1,1′-[ethane-1,2-diylbis(azan-1-yl-1-yl­idene)]bis­(ethan-1-yl-1-yl­idene)}di­pyrazine 1-oxide ligand and two mutually *trans* monodentate nitrate anions in a distorted o­cta­hedral geometry. The lattice water mol­ecule is located on a twofold rotation axis. The complex mol­ecules are linked by the water mol­ecules through O—H⋯O hydrogen bonds into a chain along [001]. Further C—H⋯O hydrogen bonds lead to the formation of a three-dimensional network.

## Related literature

For background to complexes with heterocyclic aromatic *N*-oxide ligands, see: Chupakhin *et al.* (2011 ▶); Karayannis *et al.* (1973 ▶); Nizhnik *et al.* (2008 ▶); Sarma *et al.* (2010 ▶). For related structures, see: Banerjee *et al.* (2004 ▶); Padhi & Manivannan (2007 ▶).
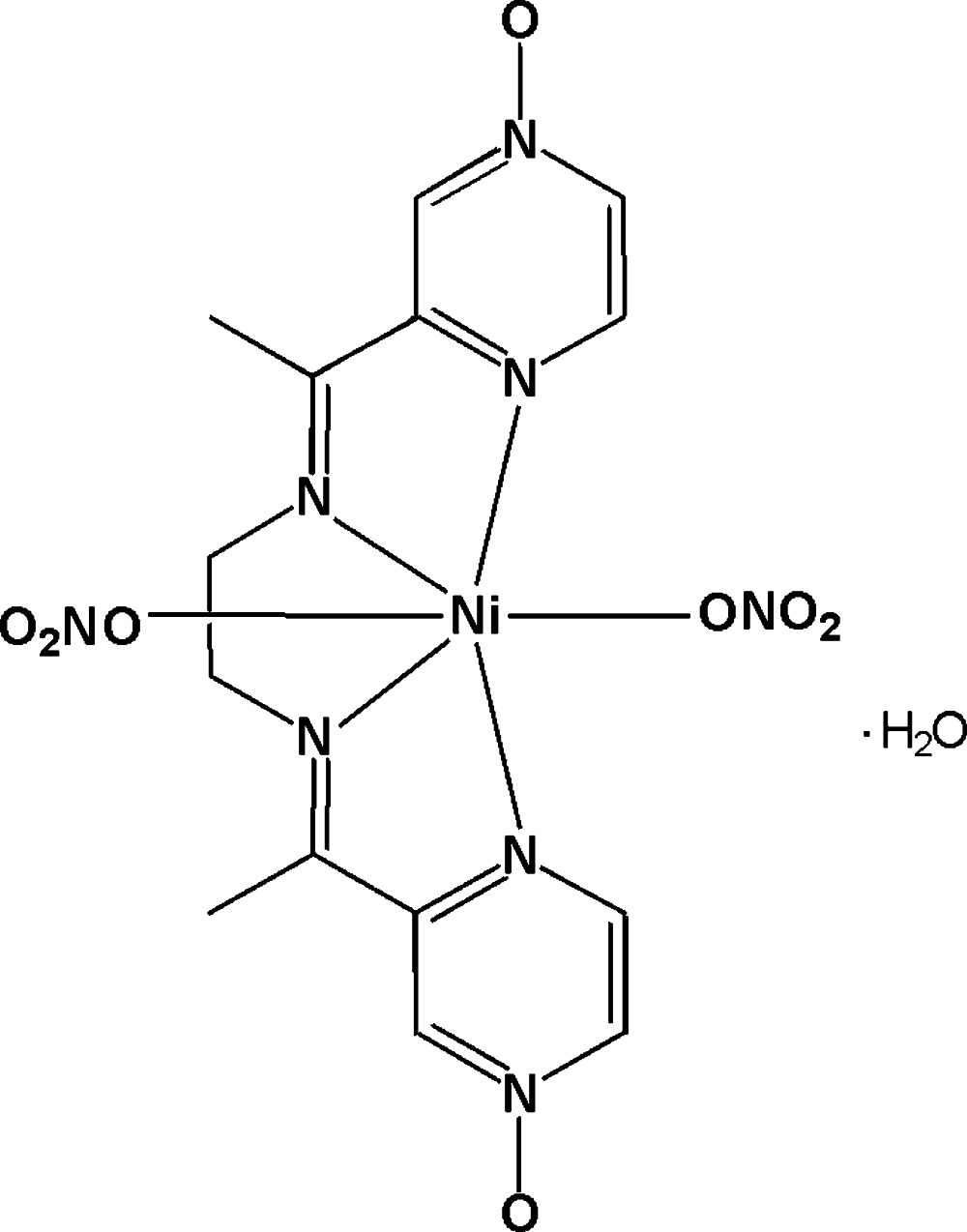


## Experimental

### 

#### Crystal data

[Ni(NO_3_)_2_(C_14_H_16_N_6_O_2_)]·H_2_O
*M* *_r_* = 501.05
Monoclinic, 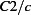

*a* = 16.993 (5) Å
*b* = 16.218 (5) Å
*c* = 7.754 (2) Å
β = 113.427 (3)°
*V* = 1960.8 (10) Å^3^
*Z* = 4
Mo *K*α radiation
μ = 1.06 mm^−1^
*T* = 293 K
0.23 × 0.21 × 0.19 mm

#### Data collection

Bruker APEXII CCD diffractometer
Absorption correction: multi-scan (*SADABS*; Sheldrick, 1996 ▶) *T*_min_ = 0.793, *T*_max_ = 0.824
6930 measured reflections
1828 independent reflections
1515 reflections with *I* > 2σ(*I*)
*R*_int_ = 0.034

#### Refinement

*R*[*F* ^2^ > 2σ(*F* ^2^)] = 0.032
*wR*(*F* ^2^) = 0.070
*S* = 1.04
1828 reflections
151 parameters
1 restraint
H atoms treated by a mixture of independent and constrained refinement
Δρ_max_ = 0.26 e Å^−3^
Δρ_min_ = −0.23 e Å^−3^

### 

Data collection: *APEX2* (Bruker, 2007 ▶); cell refinement: *SAINT* (Bruker, 2007 ▶); data reduction: *SAINT*; program(s) used to solve structure: *SHELXS97* (Sheldrick, 2008 ▶); program(s) used to refine structure: *SHELXL97* (Sheldrick, 2008 ▶); molecular graphics: *DIAMOND* (Brandenburg, 1999 ▶); software used to prepare material for publication: *SHELXTL* (Sheldrick, 2008 ▶).

## Supplementary Material

Crystal structure: contains datablock(s) I, global. DOI: 10.1107/S1600536813009355/hy2621sup1.cif

Structure factors: contains datablock(s) I. DOI: 10.1107/S1600536813009355/hy2621Isup2.hkl

Additional supplementary materials:  crystallographic information; 3D view; checkCIF report

## Figures and Tables

**Table 1:** Hydrogen-bond geometry (Å, °)

*D*—H⋯*A*	*D*—H	H⋯*A*	*D*⋯*A*	*D*—H⋯*A*
O5—H1*W*⋯O2^i^	0.78 (12)	2.46 (14)	3.086 (4)	139 (14)
C3—H3*A*⋯O2^ii^	0.93	2.58	3.389 (3)	146
C6—H6*A*⋯O1^iii^	0.96	2.56	3.453 (4)	156

Symmetry codes: (i) 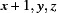; (ii) 
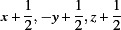
; (iii) 
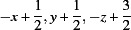
.

**Fig. 1.**
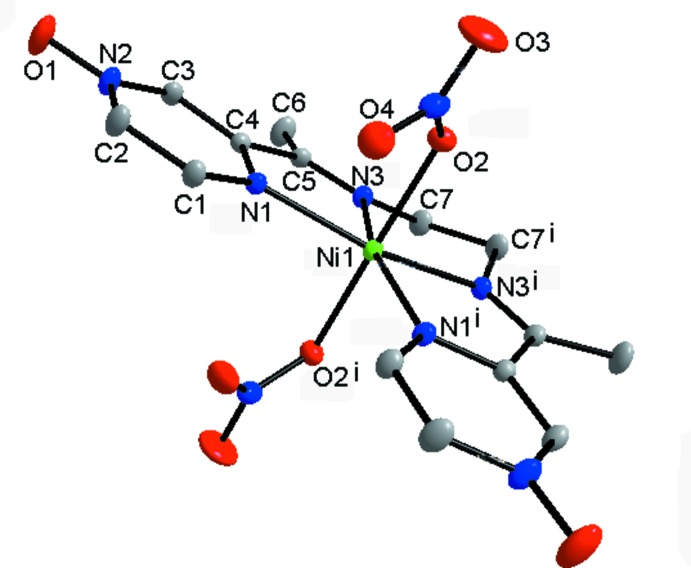
The molecular structure of the title complex. Displacement ellipsoids are drawn at the 30% probability level. H atoms and water molecule have been omitted for clarity. [Symmetry code: (i) -x, y, -z+1/2.]

**Fig. 2.**
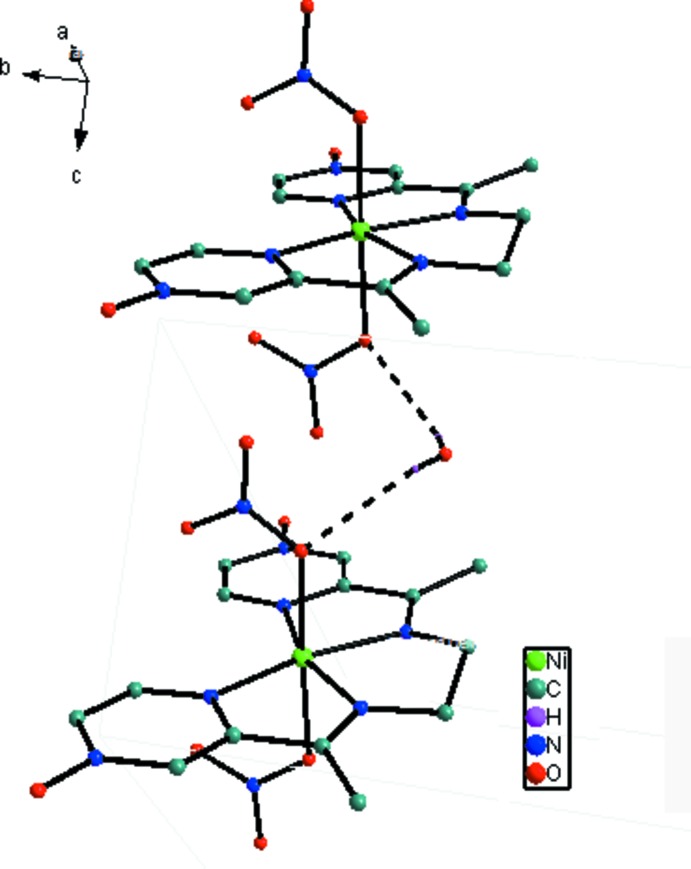
: O—H···O hydrogen bonds (dashed lines) between water molecule and two adjacent complex molecules, which lead to a chain along [001]. H atoms not involved in hydrogen bonds are omitted for clarity.

**Fig. 3.**
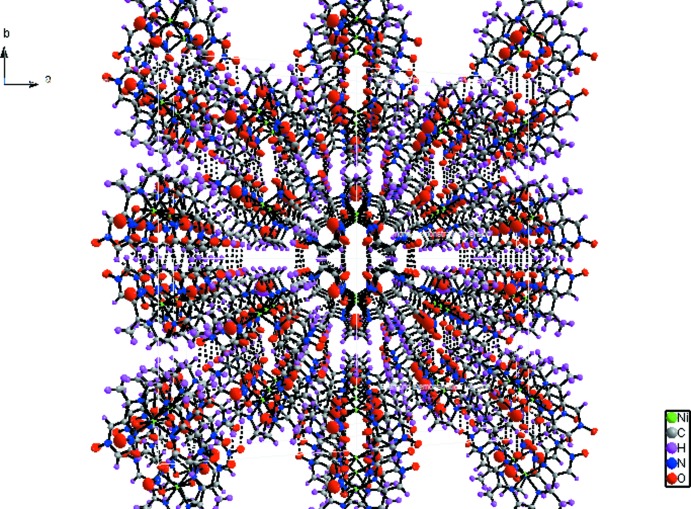
The crystal packing of the title complex, viewed along the c axis. Hydrogen bonds are shown as dashed lines.

## supplementary crystallographic information

### Comment

Interest in heterocyclic aromatic N-oxides has flourished because of their practical impact on biological activity (Sarma *et al.*, 2010). Several aromatic N-oxide derivatives capable of forming biologically active metal complexes have been reported (Nizhnik *et al.*, 2008). Indeed, most coordinative studies of N-oxide donors have been carried out using pyridine-*N*-oxide (Karayannis *et al.*, 1973) or bipyridine-*N*,*N*'-bisoxide. However, complexes containing ligands with pyrazine single N-oxide site are few (Chupakhin *et al.*, 2011). Herein, we synthesize a new bipyrazine N-oxide Schiff base 3,3'-(1E,1'E)-1,1'-[ethane-1,2-diylbis(azan-1-yl-1-ylidene)] bis(ethan-1-yl-1-ylidene)dipyrazine-1-oxide (*L*) and its Ni(II) complex.

In the title complex, the Ni^II^ atom, lying on a twofold rotation axis, exhibits a distorted octahedral geometry, defined by four N atoms from the *L* ligand, occupying the equatorial plane, and two axial O atoms from two monodentate nitrate anions (Fig. 1). The equatorial Ni—N distances [Ni1—N1 = 2.0855 (19) and Ni1—N3 = 2.0183 (19) Å] are in a normal range for this class of compounds and also very similar to those of Ni—N(pyridine) and Ni—N(imine) found in analogue complexes (Banerjee *et al.*, 2004; Padhi & Manivannan, 2007). Hydrogen bonding plays an important role in the formation of the crystal structure (Table 1). The lattice water molecule is located on a twofold rotation axis and connect two symmetry-related complex molecules through O—H···O hydrogen bonds, so forming a chain structure along [001] (Fig. 2). C—H···O hydrogen bonds lead to a three-dimensional network (Fig. 3).

### Experimental

Synthesis of 2-acetylpyrazine-N-oxide: 2-Acetylpyrazine (12.2 g, 0.1 mol), glacial acetic acid (75 ml) and 30% hydrogen peroxide (14 ml) were heated with reflux at 70–80°C for 3 h. Additional 30% H_2_O_2_ (10 ml) was added and the temperature was maintained at 70–80°C for a further 10 h. The solvent was removed by rotary evaporation and upon standing brown yellow solid was formed, filtered and dried.

Synthesis of the *L* ligand : 1,2-Diaminoethane (0.100 g, 1.66 mmol) in 5 cm^3^ methanol was added dropwise to a hot stirred solution of 2-acetylpyrazine-N-oxide (0.455 g, 3.3 mmol) in 25 ml of methanol. The mixture was refluxed for 5 h. Brown precipitate was filtered, washed with methanol and air dried (yield: 0.68 g, 68.7%).

Synthesis of the title complex: Ni(NO_3_)_2_.6H_2_O (0.290 g, 0.1 mmol) in 5 cm^3^ of CH_3_CN was added dropwise to a hot stirred solution of the ligand *L* (0.030 g, 0.1 mmol) in a mixture of CH_3_OH/CH_3_CN (*v*/*v* = 2:1) and the mixture was stirred for 30 min. Diethyl ether was slowly diffused into the solution and block brown crystals suitable for X-ray diffraction analysis were collected by filtration within two weeks.

### Refinement

C-bound H atoms were positioned geometrically and refined as riding atoms, with C—H = 0.93 (aromatic), 0.97 (CH_2_) and 0.96 (CH_3_) Å and with *U*_iso_(H)= 1.2(1.5 for methyl)*U*_eq_(C). The water H atom was located on a difference Fourier map and refined isotropically.

### Crystal data

**Table:**

[Ni(NO_3_)_2_(C_14_H_16_N_6_O_2_)]·H_2_O	*F*(000) = 1032
*M**_r_* = 501.05	*D*_x_ = 1.697 Mg m^−^^3^
Monoclinic, *C*2/*c*	Mo *K*α radiation, λ = 0.71073 Å
Hall symbol: -C 2yc	Cell parameters from 2097 reflections
*a* = 16.993 (5) Å	θ = 2.5–23.4°
*b* = 16.218 (5) Å	µ = 1.06 mm^−^^1^
*c* = 7.754 (2) Å	*T* = 293 K
β = 113.427 (3)°	Block, brown
*V* = 1960.8 (10) Å^3^	0.23 × 0.21 × 0.19 mm
*Z* = 4	

### Data collection

**Table:**

Bruker APEXII CCD diffractometer	1828 independent reflections
Radiation source: fine-focus sealed tube	1515 reflections with *I* > 2σ(*I*)
Graphite monochromator	*R*_int_ = 0.034
φ and ω scans	θ_max_ = 25.5°, θ_min_ = 2.5°
Absorption correction: multi-scan (*SADABS*; Sheldrick, 1996)	*h* = −20→20
*T*_min_ = 0.793, *T*_max_ = 0.824	*k* = −19→18
6930 measured reflections	*l* = −9→9

### Refinement

**Table:**

Refinement on *F*^2^	Primary atom site location: structure-invariant direct methods
Least-squares matrix: full	Secondary atom site location: difference Fourier map
*R*[*F*^2^ > 2σ(*F*^2^)] = 0.032	Hydrogen site location: inferred from neighbouring sites
*wR*(*F*^2^) = 0.070	H atoms treated by a mixture of independent and constrained refinement
*S* = 1.04	*w* = 1/[σ^2^(*F*_o_^2^) + (0.0282*P*)^2^ + 1.9388*P*] where *P* = (*F*_o_^2^ + 2*F*_c_^2^)/3
1828 reflections	(Δ/σ)_max_ < 0.001
151 parameters	Δρ_max_ = 0.26 e Å^−^^3^
1 restraint	Δρ_min_ = −0.23 e Å^−^^3^

### Special details

**Table:**

Geometry. All e.s.d.'s (except the e.s.d. in the dihedral angle between two l.s. planes) are estimated using the full covariance matrix. The cell e.s.d.'s are taken into account individually in the estimation of e.s.d.'s in distances, angles and torsion angles; correlations between e.s.d.'s in cell parameters are only used when they are defined by crystal symmetry. An approximate (isotropic) treatment of cell e.s.d.'s is used for estimating e.s.d.'s involving l.s. planes.
Refinement. Refinement of *F*^2^ against ALL reflections. The weighted *R*-factor *wR* and goodness of fit *S* are based on *F*^2^, conventional *R*-factors *R* are based on *F*, with *F* set to zero for negative *F*^2^. The threshold expression of *F*^2^ > σ(*F*^2^) is used only for calculating *R*-factors(gt) *etc*. and is not relevant to the choice of reflections for refinement. *R*-factors based on *F*^2^ are statistically about twice as large as those based on *F*, and *R*- factors based on ALL data will be even larger.

### Fractional atomic coordinates and isotropic or equivalent isotropic displacement parameters (Å^2^)

**Table:**

	*x*	*y*	*z*	*U*_iso_*/*U*_eq_	
Ni1	0.0000	0.22687 (3)	0.2500	0.02616 (14)	
C1	0.11963 (16)	0.08324 (15)	0.4745 (4)	0.0364 (6)	
H1	0.0798	0.0464	0.3947	0.044*	
C2	0.19290 (17)	0.05229 (17)	0.6107 (4)	0.0461 (7)	
H2	0.2022	−0.0043	0.6214	0.055*	
C3	0.23627 (16)	0.18666 (15)	0.7095 (3)	0.0352 (6)	
H3A	0.2760	0.2238	0.7884	0.042*	
C4	0.16163 (14)	0.21473 (14)	0.5717 (3)	0.0282 (5)	
C5	0.13925 (15)	0.30501 (15)	0.5473 (3)	0.0308 (5)	
C6	0.19245 (18)	0.36564 (17)	0.6900 (4)	0.0528 (8)	
H6A	0.1707	0.4202	0.6515	0.079*	
H6B	0.2507	0.3625	0.7015	0.079*	
H6C	0.1902	0.3533	0.8091	0.079*	
C7	0.03112 (17)	0.40247 (15)	0.3526 (4)	0.0397 (6)	
H7A	0.0746	0.4444	0.3731	0.048*	
H7B	0.0006	0.4150	0.4319	0.048*	
H1W	0.961 (7)	0.337 (9)	0.670 (19)	0.65 (10)*	
N1	0.10253 (12)	0.16371 (12)	0.4506 (3)	0.0289 (5)	
N2	0.25192 (14)	0.10433 (13)	0.7301 (3)	0.0437 (6)	
N3	0.07187 (12)	0.32112 (11)	0.4021 (3)	0.0302 (5)	
N4	−0.08220 (14)	0.16354 (17)	0.4974 (3)	0.0462 (6)	
O1	0.32056 (13)	0.07731 (13)	0.8616 (3)	0.0739 (7)	
O2	−0.06595 (11)	0.22970 (11)	0.4291 (2)	0.0413 (4)	
O3	−0.11896 (19)	0.16980 (18)	0.6043 (4)	0.0997 (10)	
O4	−0.06439 (14)	0.09594 (13)	0.4506 (3)	0.0603 (6)	
O5	1.0000	0.3575 (3)	0.7500	0.0993 (13)	

### Atomic displacement parameters (Å^2^)

**Table:**

	*U*^11^	*U*^22^	*U*^33^	*U*^12^	*U*^13^	*U*^23^
Ni1	0.0253 (2)	0.0206 (2)	0.0280 (2)	0.000	0.00583 (18)	0.000
C1	0.0348 (14)	0.0261 (14)	0.0407 (14)	−0.0021 (11)	0.0071 (12)	0.0025 (11)
C2	0.0413 (16)	0.0265 (14)	0.0576 (18)	0.0033 (12)	0.0059 (14)	0.0085 (13)
C3	0.0284 (14)	0.0325 (14)	0.0376 (14)	−0.0053 (11)	0.0055 (11)	0.0030 (11)
C4	0.0262 (12)	0.0287 (13)	0.0288 (12)	−0.0019 (10)	0.0100 (10)	0.0012 (10)
C5	0.0292 (14)	0.0282 (13)	0.0336 (13)	−0.0052 (11)	0.0110 (11)	−0.0025 (10)
C6	0.0490 (18)	0.0387 (17)	0.0501 (17)	−0.0044 (14)	−0.0021 (14)	−0.0097 (14)
C7	0.0454 (17)	0.0208 (13)	0.0458 (15)	0.0011 (11)	0.0106 (13)	−0.0029 (11)
N1	0.0278 (11)	0.0226 (10)	0.0336 (11)	−0.0015 (9)	0.0092 (9)	0.0011 (9)
N2	0.0331 (13)	0.0363 (13)	0.0476 (13)	0.0022 (10)	0.0011 (11)	0.0110 (10)
N3	0.0330 (12)	0.0218 (11)	0.0330 (11)	−0.0011 (9)	0.0102 (10)	−0.0021 (9)
N4	0.0339 (13)	0.0634 (17)	0.0408 (13)	−0.0025 (12)	0.0144 (11)	0.0077 (12)
O1	0.0487 (13)	0.0509 (14)	0.0815 (16)	0.0081 (11)	−0.0169 (12)	0.0172 (12)
O2	0.0453 (11)	0.0378 (10)	0.0453 (10)	0.0033 (9)	0.0226 (9)	0.0051 (9)
O3	0.118 (2)	0.125 (2)	0.100 (2)	0.0031 (19)	0.090 (2)	0.0153 (17)
O4	0.0714 (15)	0.0405 (12)	0.0721 (15)	−0.0098 (11)	0.0319 (12)	0.0052 (11)
O5	0.126 (4)	0.113 (3)	0.068 (2)	0.000	0.047 (2)	0.000

### Geometric parameters (Å, º)

**Table:**

Ni1—N3	2.0183 (19)	C5—C6	1.488 (3)
Ni1—N1	2.0855 (19)	C6—H6A	0.9600
Ni1—O2	2.1032 (17)	C6—H6B	0.9600
C1—N1	1.334 (3)	C6—H6C	0.9600
C1—C2	1.368 (3)	C7—N3	1.468 (3)
C1—H1	0.9300	C7—C7^i^	1.521 (5)
C2—N2	1.355 (3)	C7—H7A	0.9700
C2—H2	0.9300	C7—H7B	0.9700
C3—N2	1.358 (3)	N2—O1	1.283 (3)
C3—C4	1.371 (3)	N4—O3	1.224 (3)
C3—H3A	0.9300	N4—O4	1.230 (3)
C4—N1	1.351 (3)	N4—O2	1.275 (3)
C4—C5	1.506 (3)	O5—H1W	0.780 (5)
C5—N3	1.274 (3)		
			
N3—Ni1—N3^i^	81.53 (11)	N3—C5—C4	113.8 (2)
N3—Ni1—N1^i^	160.01 (8)	C6—C5—C4	120.1 (2)
N3^i^—Ni1—N1^i^	78.71 (8)	C5—C6—H6A	109.5
N3—Ni1—N1	78.71 (8)	C5—C6—H6B	109.5
N3^i^—Ni1—N1	160.01 (8)	H6A—C6—H6B	109.5
N1^i^—Ni1—N1	121.17 (11)	C5—C6—H6C	109.5
N3—Ni1—O2^i^	90.67 (7)	H6A—C6—H6C	109.5
N3^i^—Ni1—O2^i^	87.44 (7)	H6B—C6—H6C	109.5
N1^i^—Ni1—O2^i^	91.42 (7)	N3—C7—C7^i^	109.44 (14)
N1—Ni1—O2^i^	89.81 (7)	N3—C7—H7A	109.8
N3—Ni1—O2	87.44 (7)	C7^i^—C7—H7A	109.8
N3^i^—Ni1—O2	90.67 (7)	N3—C7—H7B	109.8
N1^i^—Ni1—O2	89.81 (7)	C7^i^—C7—H7B	109.8
N1—Ni1—O2	91.42 (7)	H7A—C7—H7B	108.2
O2^i^—Ni1—O2	177.50 (10)	C1—N1—C4	116.2 (2)
N1—C1—C2	123.2 (2)	C1—N1—Ni1	130.95 (16)
N1—C1—H1	118.4	C4—N1—Ni1	112.79 (15)
C2—C1—H1	118.4	O1—N2—C2	121.5 (2)
N2—C2—C1	119.9 (2)	O1—N2—C3	120.2 (2)
N2—C2—H2	120.1	C2—N2—C3	118.3 (2)
C1—C2—H2	120.1	C5—N3—C7	125.5 (2)
N2—C3—C4	119.7 (2)	C5—N3—Ni1	118.92 (16)
N2—C3—H3A	120.2	C7—N3—Ni1	114.52 (15)
C4—C3—H3A	120.2	O3—N4—O4	121.7 (3)
N1—C4—C3	122.7 (2)	O3—N4—O2	117.7 (3)
N1—C4—C5	115.32 (19)	O4—N4—O2	120.6 (2)
C3—C4—C5	122.0 (2)	N4—O2—Ni1	121.09 (16)
N3—C5—C6	126.1 (2)		
			
N1—C1—C2—N2	0.4 (4)	C4—C3—N2—O1	−178.2 (2)
N2—C3—C4—N1	−1.4 (4)	C4—C3—N2—C2	0.8 (4)
N2—C3—C4—C5	177.5 (2)	C6—C5—N3—C7	−3.4 (4)
N1—C4—C5—N3	−7.4 (3)	C4—C5—N3—C7	174.4 (2)
C3—C4—C5—N3	173.6 (2)	C6—C5—N3—Ni1	−170.6 (2)
N1—C4—C5—C6	170.5 (2)	C4—C5—N3—Ni1	7.1 (3)
C3—C4—C5—C6	−8.5 (3)	C7^i^—C7—N3—C5	164.2 (3)
C2—C1—N1—C4	−1.0 (4)	C7^i^—C7—N3—Ni1	−28.1 (3)
C2—C1—N1—Ni1	176.96 (19)	N3^i^—Ni1—N3—C5	179.1 (2)
C3—C4—N1—C1	1.4 (3)	N1^i^—Ni1—N3—C5	170.39 (19)
C5—C4—N1—C1	−177.5 (2)	N1—Ni1—N3—C5	−3.94 (17)
C3—C4—N1—Ni1	−176.86 (18)	O2^i^—Ni1—N3—C5	−93.61 (18)
C5—C4—N1—Ni1	4.2 (2)	O2—Ni1—N3—C5	88.02 (18)
N3—Ni1—N1—C1	−178.5 (2)	N3^i^—Ni1—N3—C7	10.46 (13)
N3^i^—Ni1—N1—C1	−169.8 (2)	N1^i^—Ni1—N3—C7	1.8 (3)
N1^i^—Ni1—N1—C1	3.71 (19)	N1—Ni1—N3—C7	−172.56 (17)
O2^i^—Ni1—N1—C1	−87.8 (2)	O2^i^—Ni1—N3—C7	97.77 (17)
O2—Ni1—N1—C1	94.4 (2)	O2—Ni1—N3—C7	−80.60 (17)
N3—Ni1—N1—C4	−0.56 (15)	O3—N4—O2—Ni1	178.3 (2)
N3^i^—Ni1—N1—C4	8.2 (3)	O4—N4—O2—Ni1	−4.6 (3)
N1^i^—Ni1—N1—C4	−178.30 (17)	N3—Ni1—O2—N4	−134.41 (18)
O2^i^—Ni1—N1—C4	90.16 (15)	N3^i^—Ni1—O2—N4	144.10 (18)
O2—Ni1—N1—C4	−87.66 (15)	N1^i^—Ni1—O2—N4	65.39 (18)
C1—C2—N2—O1	178.7 (3)	N1—Ni1—O2—N4	−55.78 (18)
C1—C2—N2—C3	−0.3 (4)		

Symmetry code: (i) −*x*, *y*, −*z*+1/2.

### Hydrogen-bond geometry (Å, º)

**Table:**

*D*—H···*A*	*D*—H	H···*A*	*D*···*A*	*D*—H···*A*
O5—H1*W*···O2^ii^	0.78 (12)	2.46 (14)	3.086 (4)	139 (14)
C3—H3*A*···O2^iii^	0.93	2.58	3.389 (3)	146
C6—H6*A*···O1^iv^	0.96	2.56	3.453 (4)	156

Symmetry codes: (ii) *x*+1, *y*, *z*; (iii) *x*+1/2, −*y*+1/2, *z*+1/2; (iv) −*x*+1/2, *y*+1/2, −*z*+3/2.
